# A Data-Driven Gaussian Process Regression Model for Concrete Complex Dielectric Permittivity Characterization

**DOI:** 10.3390/s25206350

**Published:** 2025-10-14

**Authors:** Giovanni Angiulli, Mario Versaci, Pietro Burrascano, Filippo Laganá

**Affiliations:** 1 Department of Information Engineering, Infrastructures and Sustainable Energy, Mediterranea University, 89121 Reggio Calabria, Italy; giovanni.angiulli@unirc.it; 2 Department of Civil, Energetic, Environmental and Material Engineering, Mediterranea University, 89121 Reggio Calabria, Italy; mario.versaci@unirc.it; 3 Dipartimento di Ingegneria—Università di Perugia, 05100 Terni, Italy; pietro.burrascano@unipg.it; 4 Laboratory of Biomedical Applications Technologies and Sensors (BATS), Department of Health Science, Magna Græcia University, 88100 Catanzaro, Italy

**Keywords:** concrete, permittivity, Gaussian processes, data-driven modeling

## Abstract

Concrete diagnosis is an important task in making informed decisions about reconstructing or repairing buildings. Among the different approaches for evaluating its characteristics, methods based on electromagnetic waves have been proposed in the literature over the years. In this context, the characterization of concrete complex dielectric permittivity $\epsilon_{r}\left(f\right)$ (where *f* is the frequency) has received considerable attention, taking into account that its values and its frequency behavior are both sensitive to a series of physical parameters, which in turn can significantly influence the mechanical performance of concrete. Recently, data-driven techniques have emerged as alternatives for modeling material properties due to their regression and generalization potential. Following this research line in this work, we investigated the potential of Gaussian Process Regression to model $\epsilon_{r}\left(f\right)$ by comparing its performance with that of the model most employed to characterize the concrete dielectric permittivity: the universal Jonscher model. The inherent ability to provide predictions accompanied by confidence intervals, which allows the assessment of the reliability of the permittivity estimate across frequency, and the related error metrics demonstrate that GPR can effectively characterize $\epsilon_{r}\left(f\right)$ in an effective manner, outperforming the Jonscher model in terms of accuracy in all the cases considered in our study.

## 1. Introduction

Due to environmental factors and dynamic loads, building structures are vulnerable to damage [1,2]. In ordinary cement concrete, cracks are inevitable due to several natural or manufactured factors, resulting in a decline in compressive strength performance and leading to structural deformation, which affects the structural integrity [2]. Accordingly, concrete diagnosis results are of primary importance for practitioners in the field in making informed decisions about reconstructing or repairing buildings [3]. During the decades, monitoring concrete mechanical properties has become an area of cross-disciplinary research, and numerous non-destructive testing evaluation (NDT) methods have been developed for this aim [1,3,4].

Alongside techniques using elastic waves, methods based on electromagnetic waves have gained popularity over the years [5]. In fact, electromagnetic techniques offer several advantages over other methods, such as the possibility of real-time monitoring of concrete building structures using contactless and wireless techniques [6,7,8], as well as the ability to achieve in situ measurements [9]. The increasing reliance on the electromagnetic approach for monitoring the properties of concrete is shaped by numerous studies published in the literature that have demonstrated the existence of relationships between electromagnetic field-related quantities and concrete properties, such as water content, porosity, and the presence of chloride [10]. Taking into account the scenario discussed so far, it becomes evident how the characterization of the concrete dielectric permittivity turns out to be essential in modeling its electromagnetic behavior as well as in retrieving its mechanical properties, such as compressive strength, which is the most important parameter to describe concrete mechanical performance [11]. To this end, researchers in the field of non-destructive testing (NDT) have placed particular emphasis on developing sensors and measurement techniques that accurately reconstruct complex permittivity through the precise measurement of concrete scattering parameters [12,13,14,15].

In the literature, various dielectric models have been employed over the years to characterize the permittivity of concrete, including the Debye model, the complex refractive index method (CRIM), the first-order exponential model, and the Cole–Cole model [16,17]. Among these, the Jonscher universal dielectric permittivity model is the one that finds the most favor among scholars and professionals in the field [1,18,19]. Recently, it was employed to evaluate the concrete shielding effectiveness, yielding notable results compared to other models, and to characterize the dielectric permittivity of Chinese standard concrete, the most widely produced type of concrete in the world [20]. Although the above models are well-established, we note that data-driven techniques have recently gained traction as alternatives for building models for property prediction and material design, owing to their ability to regress and generalize [21]. Researchers have achieved a series of milestones in developing data-driven methods that can predict electromagnetic properties with high accuracy, yielding results in excellent agreement with experimental measurements. In particular, Gaussian Process Regression (GPR) has been extensively applied to this end [22,23]. Gaussian process regression (GPR) is a powerful non-parametric supervised learning method widely used for regression and probabilistic classification tasks that, unlike the classical parametric models, defines a distribution over functions and derives predictions directly from the observed data [24]. This property makes Gaussian process regression suitable in all those situations where the underlying relationship between input and output variables is not easily expressed through analytical models, as is often the case in characterizing material properties [22,23]. A key advantage of GPR is that it provides not only point estimates, but also confidence intervals around the predictions, which are derived directly from the posterior variance of the Gaussian process and thus quantify the uncertainty associated with the prediction, a feature that distinguishes GPR from deterministic regression methods, by offering a means of assessing the reliability of the estimated function and providing a measure of confidence in the predictions, which is essential for non-destructive testing applications, where measurement errors and variability in material samples are inevitable. Based on the above, and taking into account that, to the best of our knowledge, no study has been devoted to employing GPR to characterize the complex permittivity of concrete until now, in this work, we investigated the GPR ability to describe the concrete complex dielectric permittivity $\epsilon_{r}\left(\omega \right)$ by comparing its performance with that of the universal Jonscher model to evaluate its capability to overcome the Jonscher model limitations [25]. The paper is organized as follows: Section 2 illustrates the materials and the methods employed in our analysis; in particular, Section 2.1 contains the basics on Jonscher’s universal model, while Section 2.2 and Section 2.3 discuss the foundation of Gaussian process regression and the selection criteria of its hyperparameters. Section 2.4 briefly introduces the error metrics adopted in our analysis. In Section 3, the implementation of the data-driven GPR model used to characterize the concrete complex permittivity and the related numerical performance on experimental data compared with those offered by the Jonsher model is discussed. Finally, in Section 4, we draw together some considerations and future perspectives connected with the analysis we conducted.

## 2. Materials and Methods

### 2.1. The Jonscher Universal Model

The rationale behind Jonscher’s universal dielectric model is based on empirical observations rooted in the identification of commonalities in frequency-dependent dielectric behavior conducted over a wide variety of materials differing in chemical composition and structural characteristics as well as over different frequency ranges [26]. Accordingly, the Jonscher model provides a basis for describing the frequency-dependent behavior of the complex dielectric permittivity, ϵ(ω), in matter with remarkable consistency. More precisely, the Jonscher model describes the dielectric behavior of materials by modeling relaxation processes that occur across different time scales with a power-law model, replacing the distribution of relaxation times used in Debye and Cole–Cole models with the concept of a frequency-independent ratio of energy lost to energy stored per cycle. It can be formally introduced starting from the relationship between the dielectric displacement field D and the electric field E in an isotropic solid material [26], written as follows:

(1) $$D=\epsilon \left(\omega \right)E=\left(\epsilon_{0}\chi \left(\omega \right)+\epsilon_{\infty }\right)E$$

where $\epsilon_{0}$ is the permittivity of the free space, $\epsilon_{\infty }$ is the ϵ(ω) high-frequency limit, χ(ω) is the complex electric susceptibility, and ω=2πf is the angular frequency. As said above, Jonscher derived his key insight from the experimental observations that an extensive range of solid dielectrics have a universal behavior in their susceptibility described by [26]:

(2) $$\chi \left(\omega \right)\propto \omega^{n-1}$$

where the exponent n∈[0,1] controls the material frequency dispersion behavior. The result (2) in conjunction with the causality principle, led Jonscher to conclude that the ratio between Re[χ(ω)], and Im[χ(ω)] must result independent from frequency and equal to the following:

(3) Im[χ(ω)]Re[χ(ω)]=cotnπ2.

Selecting for the real part the following functional form [26], we obtain the following:

(4) Re[χ(ω)]=χrωωtn−1

with $\omega_{t}=2\pi f_{t}$, where $f_{t}$ is the so called reference frequency [26] (which can be arbitrarily chosen within the considered frequency band [25,27]), Jonscher obtained the following expression for dielectric permittivity $\epsilon_{r}\left(f\right)$ [26]:

(5) $$\epsilon_{r}\left(f\right)=\chi_{r}{\left(\frac{f}{f_{t}}\right)}^{n-1}\left[1-i\cot\left(\frac{n\pi }{2}\right)\right]+{\bar{\epsilon }}_{\infty }$$

where $\chi_{r}$ is the value of the real part of χ(2πf) for $f=f_{t}$ and ${\bar{\epsilon }}_{\infty }=\epsilon_{\infty }/\epsilon_{0}$.

### 2.2. Gaussian Process Regression

The aim of the Gaussian Process Regression (GPR) is to exploit a set ${\left\{x_{i},y_{i}\right\}}_{i=1}^{n}$, where $x_{i}\in R^{d}$ are the data sites and $y_{i}\in R$ are the data values, for obtaining an estimator that can predict the value of the function $y_{\beta }=y\left(x_{\beta }\right)$ in correspondence to $x_{\beta }$, $x_{\beta }\notin {\left\{x_{i}\right\}}_{i=1}^{n}$, quantifing the expected error [28]. The fundamental assumption underlying the GPR approach is that the data values $y_{i}$ are the realizations of a particular random field, $\bar{Y}$, named Gaussian random process, thus defined as follows [24,28]:

**Definition** **1.** *A set of random variables is calculated as follows:*

(6) $$\bar{Y}=\left\{Y(x,\xi ),\;x\in \Omega \;and\;\xi \in W\right\}$$

*where W is a probability space, and Ω is a set called parameter space, is named as random field. If, for any arbitrarily choice of the set ${\left\{x_{i}\right\}}_{i=1}^{n}$, the vector is calculated as follows:*

$$Y=\left[\begin{array}{c} Y_{x_{1}}\left(\xi \right)\\ \vdots \\ Y_{x_{n}}\left(\xi \right) \end{array}\right]$$

*is characterized by a multivariate Gaussian distribution, with mean*

(7) $$\begin{array}{cc} \mu & =0 \end{array}$$

*and covariance*

(8) $$\begin{array}{cc} \mathrm{Cov}[Y_{x}\left(\xi \right),Y_{x^{\prime }}\left(\xi \right)] & =\sigma^{2}K \end{array}$$

*the random field $\bar{Y}$ is named Gaussian random process, where $\sigma^{2}$ is the process variance and K, defined as follows:*

(9) $$K={\left[\exp(-\varepsilon^{2}||x_{i}-x_{j}|{|}^{2})\right]}_{i,j\in \{1,\ldots ,n\}}$$

*is the covariance kernel matrix, and $\varepsilon^{2}=1/\left(2l^{2}\right)$, is a kernel-specific parameter inversely related to the square of the characteristic length-scale l, respectively.*

The random field $\bar{Y}$ can be used for regression as follows; let ${\bar{Y}}_{x}$, written as follows:

(10) $${\bar{Y}}_{x}={w(x)}^{t}\cdot \bar{Y}$$

a linear predictor belonging to $\bar{Y}$, where ${w(x)}^{t}$ are the unknowns weight functions values $w_{i}\left(\cdot \right)$ assumed in x, which must be determined through the minimization of the mean-squared error of ${\bar{Y}}_{x}$ defined as follows:

(11) $$\epsilon_{{\bar{Y}}_{x}}=E\left[{\left(Y_{x}-{w(x)}^{t}\cdot \bar{Y}\right)}^{2}\right]$$

Because of the stationarity of $\bar{Y}$, it results that $\sigma^{2}K\left(x,x^{\prime }\right)=E\left[{\bar{Y}}_{x}{\bar{Y}}_{x^{\prime }}\right]$. Using this last relation in (11), we obtain the following:

(12) $$\begin{array}{cc} \epsilon_{{\bar{Y}}_{x}}=\sigma^{2}K\left(x,x^{\prime }\right) & -2{w(x)}^{t}\left(\sigma^{2}k\left(x\right)\right)+\\ & +{w(x)}^{t}\cdot \sigma^{2}K\cdot w(x) \end{array}$$

where $k\left(x\right)={\left[K\left(x,x_{1}\right),\ldots ,K\left(x,x_{N}\right)\right]}^{T}$. To find the optimal weight vector w(x), we take the derivative of (12), with respect to w(x), and set it to zero:

(13) $$\frac{\partial \epsilon_{{\bar{Y}}_{x}}}{\partial w(x)}=\sigma^{2}\left(-2k(x)+2Kw(x)\right)=0$$

Finally, solving for w(x), we obtain the following stationary point for $\epsilon_{{\bar{Y}}_{x}}$:

(14) $$w\left(x\right)=K^{-1}\cdot k\left(x\right)$$

Using (14) in (10), and fixing the value of $\varepsilon^{2}$, the following realization of the predictor ${\bar{Y}}_{x}$ is obtained:

(15) $${\bar{y}}_{x}=k{\left(x\right)}^{t}\cdot K^{-1}\cdot \bar{y}$$

which provides the GPR regression value in correspondence of x [24].

### 2.3. Hyperparameters Selection

The generalization capability and the predictive accuracy of the GPR model are contingent upon the appropriate selection of the process variance $\sigma^{2}$, the kernel-specific parameter $\varepsilon^{2}$, which governs the covariance kernel matrix K defined by (9), and the noise variance $\sigma_{n}^{2}$, which accounts for the measurement uncertainty in the observed data. To optimize these terms, named hyperparameters, the marginal likelihood of the observed data ${\left\{x_{i},y_{i}\right\}}_{i=1}^{n}$ must be maximized. This approach leverages the nature of the random process, where the log marginal likelihood function for the zero-mean field $\vec{Y}$ is expressed as follows:

(16) $$\begin{array}{cc} \log p(y|X,\varepsilon^{2},\sigma^{2},\sigma_{n}^{2})= & -\frac{1}{2}y^{T}{\left(\sigma^{2}K+\sigma_{n}^{2}I\right)}^{-1}y-\frac{1}{2}\log\left|\sigma^{2}K+\sigma_{n}^{2}I\right|-\frac{N}{2}\log\left(2\pi \right) \end{array}$$

where $y={\left[y_{1},\ldots ,y_{n}\right]}^{t}$ is the vector of observed values, X is the design matrix of data sites, **I** is the identity matrix, and K depends on $\varepsilon^{2}$ via (9). Gradient-based optimization methods, such as conjugate gradients or quasi-Newton algorithms, are typically employed to maximize the log likelihood (16) with respect to $\sigma^{2}$, $\varepsilon^{2}$, and $\sigma_{n}^{2}$. The optimized values $\varepsilon_{\mathrm{opt}}^{2}$, $\sigma_{\mathrm{opt}}^{2}$, and $\sigma_{n,opt}^{2}$ are subsequently substituted into the predictor defined by Equation (15) to minimize regression uncertainty and enhance out-of-sample predictions [24]. Furthermore, this optimization inherently mitigates overfitting by striking a balance between model complexity and data fidelity [24].

### 2.4. Error Metrics

To compare the performance of the Jonscher model and the GPR model with each other, the following metrics were employed: The mean-squared error (RMSE), calculated as follows:

(17) $$\mathrm{MSE}=\frac{1}{n}\sum_{i=1}^{n}{\left(\epsilon_{ri}^{mod}-\epsilon_{ri}^{meas}\right)}^{2}$$

The mean absolute percentage error (MAPE), calculated as follows:

(18) $$\mathrm{MAPE}=\frac{1}{n}\sum_{i=1}^{n}\left|\frac{\epsilon_{ri}^{mod}-\epsilon_{ri}^{meas}}{\epsilon_{ri}^{meas}}\right|\%$$

The mean absolute error (MAE), calculated as follows:

(19) $$\mathrm{MAE}=\frac{1}{n}\sum_{i=1}^{n}\left|\epsilon_{ri}^{mod}-\epsilon_{ri}^{meas}\right|$$

where *n* is the number of available measured concrete complex permittivity samples, $\epsilon_{ri}^{mod}$ and $\epsilon_{ri}^{meas}$, i∈[1,…,n], are the predicted and the measured permittivity value at the frequency $f_{i}$, respectively.

## 3. Numerical Results

To implement the data-driven GPR model the built-in functions fitrgp and predict of Matlab 2023b ^©^ Statistics and Machine Learning Toolbox and the related options have been used [29]. Algorithm 1 reports the MATLAB pseudocode of the implemented procedure. All the datasets considered in our study are of the following form:

$$D={\left\{x_{i}=f_{i},y_{i}=\epsilon_{i}^{meas}\right\}}_{i=1}^{n}$$

where the *i*-th tuple in D corresponds to the permittivity measurement $\epsilon_{i}^{meas}$ at the discrete frequency $f_{i}$ ($x_{i}=f_{i},R^{d}=R$) for a specific concrete specimen. The Jonscher parametrization for each of the cases considered in this study is reported in Table 1. The kernel function we used was specified as the squared exponential (Gaussian) kernel using the options (’KernelFunction’, ’squaredexponential’) included in the fitrgp function [29]. Furthermore, we used the option (‘Standardize’, true) available in the options of the same function to standardize the input predictors, i.e., in this case the frequency values, by removing the mean and scaling to unit variance before training, thus improving kernel resizing and optimization stability without altering the physical units of frequency or the predicted permittivity [29]. Regarding the setting of the hyperparameters ε, $\sigma^{2}$, and $\sigma_{n}^{2}$ we optimized them using the predefined options’ (Fitmethod’, ’exact’), and (’Optimizer,’ ’quasinewton’) included in the fitrgp function [29]. The ‘exact’ fit option was used due to the small size of the data sets considered, allowing for a precise calculation of the covariance matrix, while the ‘quasinewton’ optimizer was selected for its efficiency in solving the relative unconstrained optimization problem. The involved CPU time for all computations running on a machine based on Intel(R) Core(TM) i5-4570S CPU at 2.90 GHz was a few fractions of a second (Intel Corporation, Santa Clara, CA, USA). All the test and training datasets employed to build the GPR models discussed in the following were created by randomly partitioning the dataset D across frequencies.

The first case we considered involved two datasets, each composed of n=16 tuples from the experimental data reported in [27]. The first set of data was relevant to concrete characterized by a water-to-cement ratio, W/C, of 0.30, average porosity, AP, of 8.7% and bulk density, BD, of 2.27 (g·cm ^−3^) labeled as B1, while the second one was relevant to concrete with a W/C of 0.66, AP equal to 13.7%, and BD of 2.18, labeled as B2. The permittivity data were collected in the frequency range [0,1] GHz [27], see Algorithm 1.
**Algorithm 1** Matlab GPR concrete permittivity modeling pseudocode.1: Input: Data_concrete, dim_training_set; 2: Output: ${\bar{y}}_{x_{Re}},{\bar{y}}_{x_{Im}}$, $\;\;\;\;MSE_{Re},MAE_{Re},MAPE_{Re},$ $\;\;\;\;MSE_{Im},MAE_{Im},MAPE_{Im};$ 3: function [$DTrain_{f},DTrain\epsilon_{rRe},DTrain\epsilon_{rIm},DTest_{f},DTest\epsilon_{rRe},DTest\epsilon_{rIm},idx$]= PartitionDATA(Data_concrete, dim_training_set); 4: GPR_Re=fitrgp(DTrainf,DTrainϵrRe); 5: GPR_Im=fitrgp(DTrainf,DTrainϵrIm); 6: y¯xRe=predict(GPR_Re,DTestf); 7: y¯xIm=predict(GPR_Im,DTestf); 8: function [$MSE_{Re},MAE_{Re},MAPE_{Re}$]=$\mathrm{ErrorMETRICS}({\bar{y}}_{x_{Re}},DTest\epsilon_{rRe})$; 9: function [$MSE_{Re},MAE_{Re},MAPE_{Re}$]=$\mathrm{ErrorMETRICS}({\bar{y}}_{x_{Im}},DTest\epsilon_{rIm})$; 10: end


**Figure 1:**
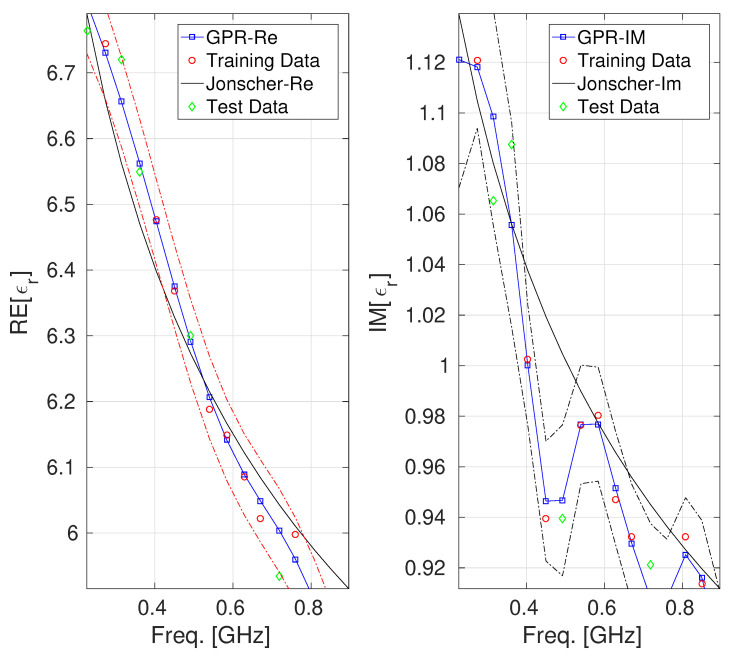
Behavior of the real and the imaginary parts of $\epsilon_{r}$ for the concrete labeled as B1 [27]: GPR model vs. Jonscher parametrization. The uncertainty bands (epsilon-tube) are also reported (identified by the red and black dashed lines).

**Figure 2:**
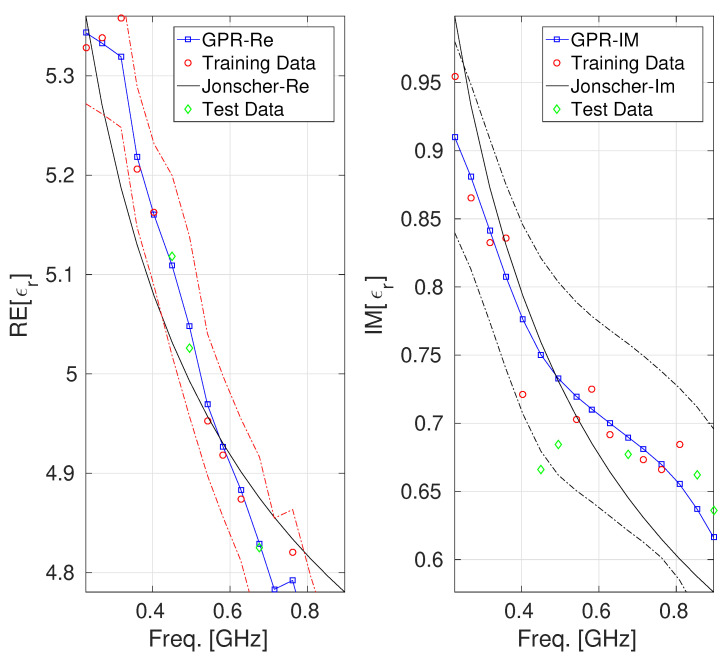
Behavior of the real and the imaginary parts of $\epsilon_{r}$ for the concrete labeled as B2 [27]: GPR model vs. Jonscher parametrization. The uncertainty bands (epsilon-tube) are also reported (identified by the red and black dashed lines).

We built up two GPR models: one for characterizing the real part and the other for the imaginary part of the complex permittivity of the concrete at hand. For both considered datasets, the set used to train the GPR model for model fitting and hyperparameter optimization consisted of 70% of the tuples belonging to D. In contrast, the pertinent test set consisted of the remaining 30 %. Figure 1 and Figure 2 report a comparison among the values of the real and the imaginary parts of $\epsilon^{meas}$ provided by the Jonscher and the GPR model. The figures also display the confidence intervals, also known as epsilon tubes, for both GPR models (represented by the dashed red and blue lines, respectively). These intervals represent the uncertainty bounds around the GPR predictions, enabling the assessment of the predictive performance and generalization capabilities of both GPR models.

Regarding the model characterizing the real part of the dielectric permittivity of concrete B1, it can be observed from the left side of Figure 1 that the confidence interval results are symmetric and not too wide, indicating a good characterization of the prediction. Furthermore, the test data all fall within the confidence interval, indicating that the model is faithful and capable of good generalization. This behavior suggests a well-calibrated GPR model for the real part of the concrete permittivity with limited overfitting and a reliable quantification of uncertainty. About the GPR model characterizing the imaginary part of the dielectric permittivity of the concrete B1 (reported on the right side of Figure 1, we can observe that the confidence interval results in a reasonable width, not too narrow (which would indicate overconfidence) and not too wide (which would indicate high uncertainty) across the frequency band, which suggests that the model is accurate enough in this range. The confidence interval related to the GPR model of the real part of the B2 concrete permittivity (which is reported on the left side of Figure 2) shows that the GPR predictions remain tightly bounded within this interval, with the test data falling entirely within the bound it defines. In contrast, the GPR model of the imaginary part of the B2 concrete permittivity exhibits a broader confidence interval, particularly for frequencies greater than 0.4 GHz (where a test point falls marginally outside the predicted confidence region). Although the GPR model reproduces the general shape of the imaginary permittivity curve, the increasing width of the uncertainty band may point to localized model underfitting or regions of sparse training data, supporting the interpretation that the model may be potentially less accurate in this area, a behavior attributable to the low number of samples composing the dataset under consideration. Table 2 and Table 3 provide a quantitative estimation of the performance offered by the different models, using MSE, MAE, and MAPE metrics, which are computed exclusively on the test set to ensure an unbiased comparison [24]. It can be observed that GPR achieves lower error values for all considered metrics.

**Figure 3:**
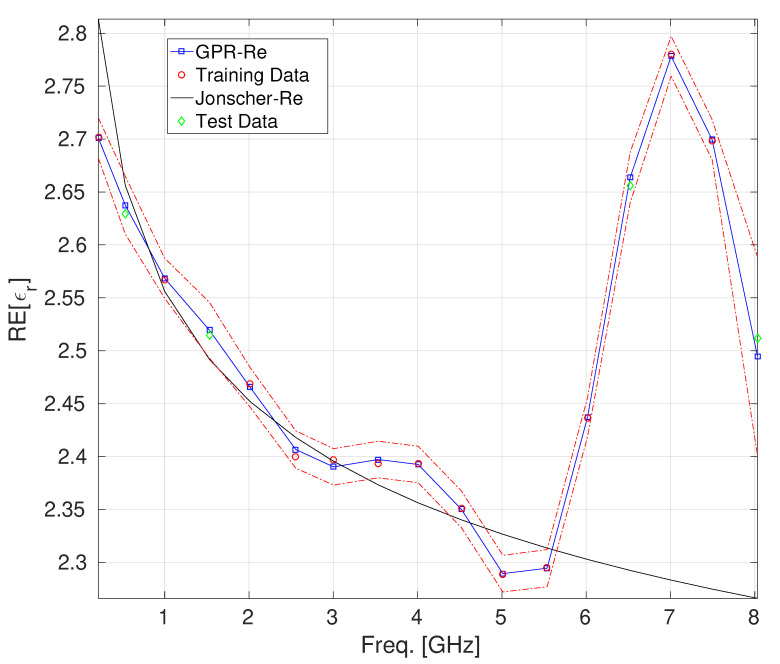
The behavior of the real part of $\epsilon_{r}$ for the lightweight foamed concrete [30]: GPR model vs. Jonscher parametrization. The uncertainty bands (epsilon-tube) are also reported (identified by the red and black dashed lines).

As a second case, we considered the dataset from [30], which pertains to lightweight foamed concrete (LFC) made of sand, foam, and ordinary Portland cement. The dataset consisted of *n* = 18 permittivity samples collected in the frequency range of [0,8] GHz, referred to as the LFC, with a density of 1368 kg/m ^3^. Since, in this case, only the values of the real part of the complex permittivity were available, a GPR model was developed to characterize this quantity alone, using 70% of the samples as the training set and the remaining 30% as the test set. Figure 3 and Table 4 report the results obtained for the considered case. The confidence interval characterizing the GPR prediction, within which all test points fall, reveals, once again, that the GPR model has a more than good ability to describe the behavior of the real part of the dielectric permittivity of concrete for the case under consideration. In particular, the superiority of the GPR characterization compared to the Jonscher one is apparent, especially in the frequency range above 5 GHz.

**Figure 4:**
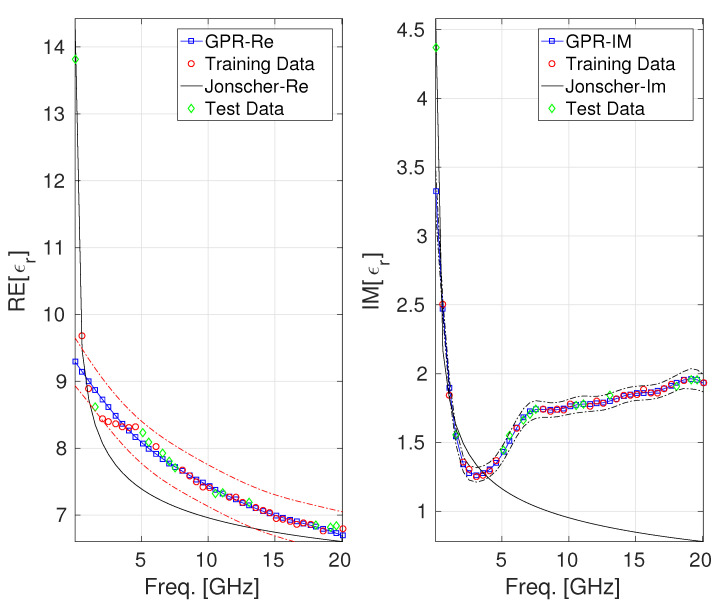
Behavior of the real and the imaginary parts of $\epsilon_{r}$ for the ultra-lightweight cement composite [25]: GPR model vs. Jonscher parametrization. The uncertainty bands (epsilon-tube) are also reported (identified by the red and black dashed lines).

The last case we considered regarded the dataset of permittivity from [25] about an ultra-lightweight cement composite (ULCC) fabricated using cement, water, fly ash, cenosphere, unoiled polyvinyl alcohol (PVA) fibers (2% volume fraction), and high-range water reducers (which are chemical admixtures used in concrete to reduce the amount of water needed while maintaining workability significantly). For this last case, the exploited dataset consisted of n=42 samples collected in the range [0,20] GHz. The two GPR models we developed were trained using 65% of the available tuples, while the remaining 35% were used to build the test set. In Figure 4, a comparison among the values of the real and the imaginary parts of $\epsilon_{r}\left(f\right)$ provided by the Jonscher and the GPR model is reported. The confidence intervals, qualitatively slightly wider in the case of GPR predictions on the real part of $\epsilon_{r}\left(f\right)$ and narrower in the case of GPR predictions on the imaginary part, demonstrate, for this last case also, the goodness of the GPR modeling. Table 5 shows a more quantitative assessment of the performance of the different models using MSE, MAE, and MAPE metrics, which reveals the superiority of the GPR modeling over the Jonscher law.

## 4. Conclusions

This work presents the results of a data-driven Gaussian Process Regression (GPR) modeling approach employed to characterize the complex dielectric permittivity $\epsilon_{r}\left(f\right)$ of concrete. The GPR models were trained on experimental datasets of concrete permittivity, and results were compared with measurements and to those provided by the well-established Jonscher universal dielectric model.

The related error metrics demonstrate that GPR can effectively characterize $\epsilon_{r}\left(f\right)$ in an effective manner, outperforming the Jonscher model in terms of accuracy in all the cases considered in our study. Furthermore, the ϵ−tubes generated by our Gaussian Process Regression models provide direct evidence against overfitting, along with a reliability assessment of the permittivity estimate across frequency. Although our study involved only three sets of data with specific compositions, and despite the small number of samples (*n* = 17, 18, 42) used for our numerical validations, limited by the data available in the literature on the subject, the three types of concrete considered (normal weight B1/B2, lightweight LFC, and ultra-lightweight ULCC) represent strategically different cases covering different ranges of density, composition, and frequency behavior. This peculiar characteristic of the data sets considered in our study provides strong evidence, based also on the fact that the error metrics related to the GPR model exceed those related to the Jonscher model for this spectrum of materials, of the methodological value of the proposed approach to characterize the complex permittivity of concrete.

For completeness, we would like to point out that, although we did not conduct a formal sensitivity analysis in this study, the predictive performance and superiority of GPR over the Jonscher model that we observed were found in several subdivisions of the datasets and default initialization settings, with the quasi-Newton algorithm achieving stable solutions in a few iterations (usually 7–13), thus demonstrating convergence behavior that indicates reasonable insensitivity of the GPR models we developed to specific hyperparameter values within the ranges relevant for characterizing the complex permittivity of concrete.

Finally, it should be noted that the complex permittivity of concrete $\epsilon_{r}\left(f\right)$ does not depend exclusively on the frequency alone, but also on its physical composition (W/C ratio, porosity) [17,18]. This last observation provides a starting point for future work, which will focus on integrating these physical parameters into a more advanced, complex permittivity GPR model capable of handling multiple typologies to estimate compressive strength from in situ electromagnetic measurements. Furthermore, since measurements of concrete dispersion parameters are often only available over a limited frequency band [25], a sequential data splitting strategy will be tested to create training and test sets for the GPR permittivity model, with the aim of evaluating its ability to extrapolate permittivity values beyond the training range.

## Figures and Tables

**Table 1:** Jonscher parameters for the types of concrete considered in this work (the $f_{t}$ values are also reported).

Concrete	*n*	$\chi_{r}$	${\bar{\epsilon }}_{\infty }$	$f_{t}$ (GHz)
B1	0.777	3.82	3.56	0.2266
B2	0.683	2.10	3.71	0.2266
LFC	0.901	1.55	1.0	0.2129
ULCC	0.712	3.73	5.0	0.0321

**Table 2:** Error metrics values for the $\epsilon_{r}$ characterization provided by the Jonscher and by the GPR models reported in Figure 1.

B1	MSE	MAE	MAPE
Jonscher(Re)	0.0422	0.0799	1.24
GPR(Re)	0.021	0.0398	0.622
Jonscher(Im)	0.0175	0.0352	3.45
GPR(Im)	0.0152	0.0289	2.65

**Table 3:** Error metrics values for the $\epsilon_{r}$ characterization provided by the Jonscher and by the GPR models reported in Figure 2.

B2	MSE	MAE	MAPE
Jonscher(Re)	0.0488	0.0961	2.02
GPR(Re)	0.00882	0.0167	0.349
Jonscher(Im)	0.0291	0.0614	9.26
GPR(Im)	0.0207	0.0381	5.71

**Table 4:** Error metrics values for the $\epsilon_{r}$ characterization provided by the Jonscher and by the GPR models reported in Figure 3.

LFC	MSE	MAE	MAPE
Jonscher(Re)	0.11	0.165	6.35
GPR(Re)	0.00514	0.00927	0.361

**Table 5:** Error metrics values for the $\epsilon_{r}$ characterization provided by the Jonscher and by the GPR models reported in Figure 4.

ULCC	MSE	MAE	MAPE
Jonscher(Re)	0.147	0.481	5.97
GPR(Re)	0.349	0.421	3.46
Jonscher(Im)	0.216	0.695	38.1
GPR(Im)	0.0807	0.0948	2.68

## Data Availability

Data are contained within the article.

## Abbreviations

GPR	Gaussian Process Regression;
MSE	Mean-Squared Error;
MAPE	Mean Absolute Percentage Error;
MAE	Mean Absolute Error
